# Familial Clustering and Reinfection With 2019 Novel Coronavirus (COVID-19, SARS-CoV-2) in the Libyan Community

**DOI:** 10.1017/dmp.2021.68

**Published:** 2021-03-08

**Authors:** Mohamed A. Daw, Muheeb M. Miftah, Abdallah El-Bouzedi, Mohamed O. Ahmed

**Affiliations:** 1 Department of Medical Microbiology & Immunology, Faculty of Medicine, University of Tripoli, Tripoli, Libya; 2 Faculty of Medicine, University of Tripoli, Tripoli, Libya; 3 Bani Waleed, University, Bani-Waleed, Bani-Waleed, Libya; 4 Department of Laboratory Medicine, Faculty of Biotechnology, University of Tripoli, Tripoli, Libya; 5 Department of Microbiology & Parasitology, Faculty of Veterinary Medicine, University of Tripoli, Libya

Since its emergence as a major cause of coronavirus pneumonia, severe acute respiratory syndrome coronavirus 2 (SARS-CoV-2) has spread quickly all over the world. The pandemic has affected all aspects of life and continues to spread despite the stringent control measures. Meanwhile, scientists all around the world have been scrambling to ascertain how the virus spreads and find out the effective ways to put this outbreak quickly under control, focusing on both persistent strict domestic interventions and vigilance against exogenous imported cases.^1,2^ Several cases of family clusters have been reported and evidence of person-to-person transmission has been confirmed, indicating the importance of early detection and isolation of infected patients.^3,4^ Hence, special caution is needed for asymptomatic patients, particularly for family members.

Furthermore, due to the widespread expansion of the coronavirus disease 2019 (COVID-19) epidemic around the world, different areas experienced a resurgence of COVID-19 cases after a relaxation of social distancing policies. However, there is an emerging concern regarding the reinfection in previously recovered SARS-CoV-2 patients. In former coronavirus pandemics, including the Middle East respiratory syndrome (MERS) and severe acute respiratory syndrome (SARS), immunoglobulin levels in patients lasted for a minimum of 2 years, indicating that patients could be vulnerable to reinfection after 3 years. Emerging evidence suggested that SARS-CoV-2 could present similar behavior and reactivate in patients with previously confirmed COVID-19 infection and cause illness and person-to-person transmission.^5,6^

Herein, we report on 2 epidemiological conditions that evolved during the pandemic spread of novel coronavirus (COVID-19), within the Libyan community^7,8^.

**Figure 1.**
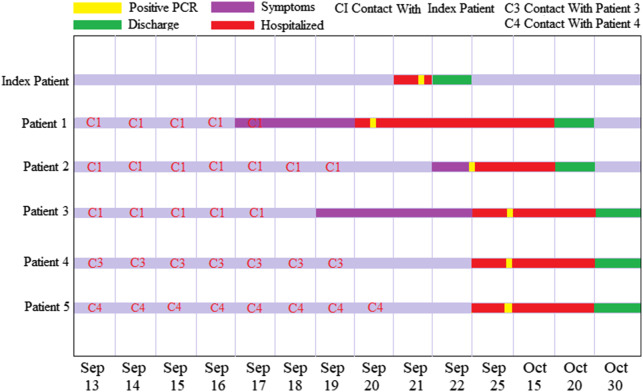
Timeline of exposure to an index patient with COVID-19 infection in the familial cluster case in Libya.

*The first condition we report concerns* the epidemiological features of a family cluster of 6 patients in Libya, as 1 of them had a history of travel to Istanbul and 5 other family members had not traveled anywhere. The demographic, epidemiological, and clinical features; chest radiography; laboratory tests; and outcomes were obtained for each patient. An asymptomatic case was defined as a laboratory-confirmed COVID-19 infection case who was afebrile and well.^7^ A total of 6 patients of the same family were transferred to a special COVID-19 center designated for treatment and isolation from September 13 to October 27 as illustrated in Figure 1. The familial cluster of 6 patients (index patient to patient 5) was infected with COVID-19, and just the index patient had been to Turkey, who had no evident symptoms before his family members started to get sick one after another. All the family members live in one house, sharing the same rooms, and having their meals together. On September 13, the index patient, a 46-year-old male, came back from Istanbul, Turkey, and stayed with his family. Afterwards, the family started to get sick. On September 17, his mother (patient 1), a 68-year-old woman, became ill with continuous high fever and fatigue; the highest body temperature was 40.2°C; she was admitted to the hospital on September 20. Nasopharyngeal swabs samples were obtained for this patient and were found to be positive for COVID-19 on the real-time reverse transcription-polymerase chain reaction (RT-PCR) assay. On September 22, the index patient’s brother, patient 2, 43-year-old male, had a fever of 38.1°C too. The family visited a hospital together and were tested (RT-PCR) for COVID-19, the results of which were all positive (Figure 1). Then, the whole family was transferred to the designated center for special treatment and isolated on September 25. On October 19, 2020, index patient to patient 4 were discharged: the index patient had no fever along with dry cough, patient 4 had no clinical symptoms during hospitalization. Patient 5 stayed in the hospital and then was released on October 27. All 6 family members (index patient and patients 1, 2, 3, 4, 5) tested positive for COVID-19 by RT-PCR. Three patients (patients 2, 3, 5) had associated symptoms at the time of presentation.

**Figure 2.**
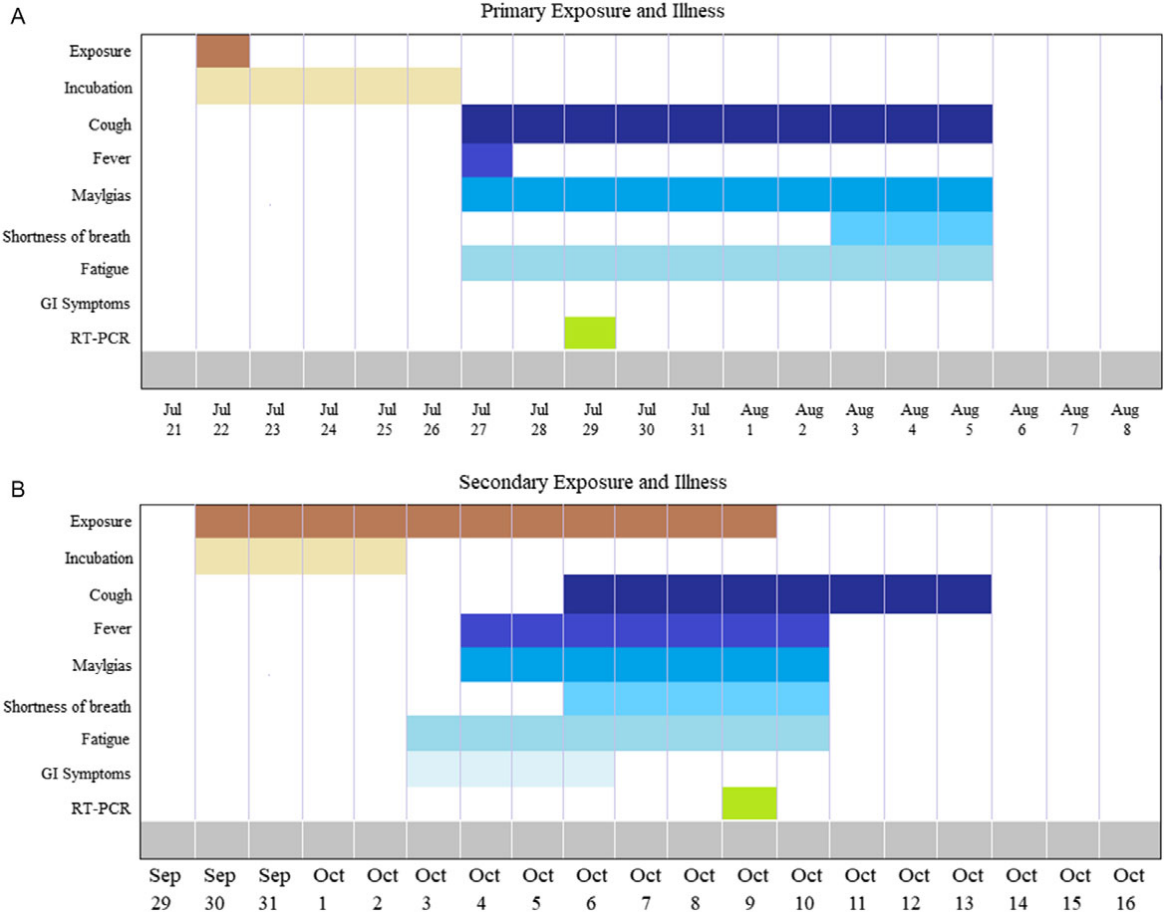
A, Timeline of primary infection indicating clinical symptoms confirmed by RT-PCR testing. B, Reinfection timeline of the same patient showing secondary clinical symptoms confirmed by RT-PCR testing.

*The second condition is regarding* a case of reinfection from SARS-CoV-2 in the Libyan Community (Figure 2). A 52-year-old healthy male residing in Libya was admitted with cough, sore throat, fever, myalgias, and headache on July 22, 2020. The diagnosis was confirmed positive by RT-PCR. As all his symptoms subsided, the patient was discharged on August 8, 2020, upon 2 negative RT-PCR assays on nasopharyngeal and throat swabs taken 24 h apart (Figure 2A).^7,8^

During the second episode, on September 29, 2020, the patient was admitted again with fever, cough, shortness of breath, and gastrointestinal symptoms (Figure 2B). The clinical and radiographic examination showed a higher temperature, blood pressure, and pulmonary infiltrate. RT-PCR assay was also positive. It is worth noting that the symptoms were significantly worse when compared with the initial episode. Despite the lack of sequencing data, the clinical and epidemiological manifestations of this case indicated early reinfection with SARS-CoV-2, only 52 d after resolution of the initial infection. This is in concordance with other published data by To *et al*. and Mulder *et al*.^9,10^ However, further studies are needed to clarify whether this second infection is due to the acquisition of a new or more pathogenic strain, particularly after the emergence of a new virulent strain nowadays.

Family clustering and reinfection represent a new clinical and epidemiological challenge in the COVID-19 pandemic. Therefore, policy planning should consider these contributing factors. Family clusters were common in the development of this epidemic. Hence, special caution is needed for asymptomatic family members, and policies should be developed to forbid large-scale family meetings and unnecessary visits. Furthermore, reinfection findings may have certain implications whether the reinfection occurred with the same stain or a newly emerging one. This highlights the role of vaccination in response to COVID-19. If we have truly reported a case of reinfection, initial exposure to SARS-CoV-2 might not result in a level of immunity that is defiantly protective for all individuals indicating similar perspectives to the influenza vaccine. However, initial genome analysis studies showed that the SARS-CoV-2 strains from the first and second episode in our patient belong to different clades/lineages with 24 nucleotide differences, suggesting that the virus strain detected in the second episode is completely different from the strain found in the first episode.^6^
